# Methodological considerations in clinical outcomes assessment of pharmacy-based minor ailments management: A systematic review

**DOI:** 10.1371/journal.pone.0205087

**Published:** 2018-10-04

**Authors:** Vibhu Paudyal, Scott Cunningham, Kathrine Gibson Smith, Katie MacLure, Cristin Ryan, Maria Cordina

**Affiliations:** 1 School of Pharmacy, Institute of Clinical Sciences, University of Birmingham, Birmingham, United Kingdom; 2 School of Pharmacy and Life Sciences, Robert Gordon University, Aberdeen, United Kingdom; 3 School of Pharmacy, Trinity College Dublin, Dublin, Republic Of Ireland; 4 Faculty of Medicine & Surgery, University of Malta, Msida, Malta; University of Waterloo, CANADA

## Abstract

**Background:**

The accessibility of services within community pharmacies provides an ideal opportunity to manage minor ailments, yet over £1.1 billion is spent by the National Health Service (NHS) in the United Kingdom (UK) in managing minor ailments in high cost settings. There is a need to review the evidence base around clinical effectiveness of pharmacy-based management of minor ailments since the absence of such may lead to under-utilisation of pharmacy services and non-implementation of available pharmacy service models. This study aimed to systematically review the methodological approaches used to assess clinical outcomes of pharmacy-based management of minor ailments in the research literature.

**Methods:**

A systematic review was conducted to identify relevant literature using the following databases: Medline, EMBASE, CINAHL, IPA, CRD, CDSR, and Google Scholar from publication year 2000 onwards. Studies were included if they evaluated clinical outcomes of pharmacy-based management of any minor ailments, with or without a comparator setting such as Emergency Departments (EDs) or general practices. Screening and selection of titles, abstracts and full texts followed by data extraction and quality assessment (QA) was conducted. Paired researchers, from the team, reviewed papers using a protocol based on the Preferred Reporting Items for Systematic Review and Meta-Analysis Protocols (PRISMA-P). QA was undertaken using the Critical Appraisal Skills Programme (CASP). Reporting was conducted in accordance with PRISMA checklist and statements.

**Results:**

A total of 19 studies were included. The majority of studies were observational, conducted in community pharmacies, and did not use a comparator participant group nor a comparator setting. Interventions included counselling, medicines supply and provision of advice on the management of minor ailments. One study used the randomised controlled trial (RCT) design with majority of the study utilising observational design. A range of clinical outcomes including symptom severity, pattern, resolution, and quality of life were reported. Methods used for the assessment of clinical outcomes were, overall, poorly reported. This included a lack of information on the development and validation of the data collection tools and the timing of baseline and follow-up data collection. Adverse clinical outcomes data were collected by only seven studies.

**Conclusions:**

Currently, there are methodological limitations in the studies that have sought to assess clinical outcomes of pharmacy-based management of minor ailments. Such lack of high quality evidence may contribute to failings to shift care from high cost settings, such as EDs and general practices. Generation of high quality evidence is likely to influence public choices when seeking care for minor ailments. There is scope for development of a core outcomes set specific to minor ailments management and development of a validated methodology for measuring such outcomes in a research study.

## Background

Minor ailments are defined as ‘common or self-limiting or uncomplicated conditions which can be diagnosed and managed with minimum or no professional support’ [1]. For example, cough, cold, hay fever, red eye, minor sprains and pains. Despite the widely acknowledged expertise of community pharmacists, and pharmacy support staff, and their contribution to the management of minor ailments, internationally, there is an under-utilisation of pharmacy. For example, a recent analysis of routinely collected data in hospital emergency departments (ED) and in general practices in the United Kingdom (UK) demonstrated that between 5% and 13% of consultations respectively were for minor ailments equating to a cost of £1.1 billion [2]. Up to 20% of all general practitioner (GP) consultations are known to be for minor ailments consultations alone excluding the data where minor ailments forms a part of a consultation for a more serious condition [3]. The services of pharmacists in ED and general practices in managing minor ailments, inclusive of the recruitment of pharmacist independent prescribers, has recently been introduced in the UK [4].

There is overwhelming support for enhanced pharmacy-based management of minor ailments from pharmacists and associated professional and regulatory bodies across Europe [5–8]. For example, the Royal Pharmaceutical Society (RPS), the pharmacy regulator in the UK, supports the enhanced management of minor ailments through community pharmacy. It is proposed that enhanced management of minor ailments from pharmacy can improve patient choices in their use of health services, reduce the burden on general practice and ED, increase patient access to local services for minor ailment management and permit professional development opportunities for pharmacists. The government policies also envisage that enhanced pharmacy assisted self-care would contribute to significant patient health benefits in the longer term [6].

The existing burden of patients presenting with minor ailments to general practice and ED highlights the need for further research to identify any gaps between policy and practice. Lack of adequate evidence may negatively impact on the promotion of relevant practice and policy implementation [9–11]. Pharmacy-based services such as the Minor Ailment Scheme (MAS), which allows free access to the management of minor ailments, have been introduced through all community pharmacy across Scotland. Patients may present with a minor ailment in a community pharmacy and expect to receive a structured, formulary-based approach to treatment from a trained, pharmacy-based team. Those who fulfil eligibility criteria such as those 60 years or older, pregnant women and those on income support and allowance, are able to obtain over–the-counter medicines at no cost, thereby increasing access to treatments for patients where affordability may be a barrier [12]. In England, however, this particular service has not been offered at a national level, and Primary Care Trusts in England are yet to implement a publicly funded minor ailments scheme in their regions. Similar models of Government funded pharmacy-based management of minor ailments are uncommon in European countries [13].

The development of a robust evidence base for pharmacy-based management of minor ailments necessitates that the strengths and limitations of the current evidence is reviewed. Types of clinical outcomes and their methods of assessment relating to pharmacy minor ailments management have not previously been systematically reviewed. Measuring clinical outcomes of minor ailment management in research evaluation presents challenges for data collection, analysis and interpretation. For example, typically short and self-limiting episodes of illness, diversity in the range of conditions considered to be minor ailments and lack of appropriate follow-up methods are inherent issues in minor ailments research. Therefore, it is often difficult in research studies, including comparative evaluations, to synthesise clinical outcomes across different illnesses, treatments and settings. Currently, there is no gold standard with regard to the type of clinical outcome data that should be assessed and the methods of assessment that should be deployed as a component of an intervention study.

This study aimed to systematically review the methods and types of clinical outcomes assessment used in the evaluation of pharmacy-based minor ailment management.

## Methods

A protocol was prepared using Preferred Reporting Items for Systematic Review and Meta-Analysis Protocols (PRISMA-P, CRD42016050847) (S1 Appendix 1) [14]. An electronic search of MEDLINE, EMBASE, CINAHL, Centre for Review and Dissemination database (CRD), Cochrane Database of Systematic Reviews (CDSR), and Google Scholar were undertaken using Medical Subject Headings (MeSH) and natural language key words, Boolean and proximity (e.g. adj2] operators, truncations (*) and wild cards ($). The draft search strategy was utilised to perform a scoping search and moderated by the research team before a final version was agreed for each database. The reference lists of included studies were hand searched to identify any additional references for inclusion. Authors of conference abstracts were contacted to identify any related full text publications. Referencing software was used to manage included and excluded references and remove duplicate search results. An example search strategy is provided in S2 Appendix.

### Eligibility criteria

No restrictions to participant demography, study design or publication language were applied. Evaluations of pharmacy-based management of minor ailments, either with or without a comparator service, and from all settings were included from the publication year 2000 onwards. Studies which did not involve an assessment of follow-up clinical outcomes were excluded from the review. Both grey and peer reviewed literature were considered. Abstract only publications were excluded.

### Screening and selection

Screening was conducted by one pair of researchers in three consecutive stages: screening of titles; screening of abstracts; screening of full text against the inclusion and exclusion criteria listed in the protocol (S1 Appendix). Disagreements regarding inclusion were resolved via discussion within the pairs.

### Data extraction and quality assessment

A data extraction tool was developed based on the review aims and objectives, refined, reviewed and approved by the research team. The tool was piloted using two studies which were subsequently included in the review. The Critical Appraisal Skills Programme (CASP) quality assessment tools (for randomised controlled trials (RCT) and cohort studies) were used to assess study quality [15]. Studies were not excluded based on quality since the quality assessment of existing literature pertained to the study objectives. Data extraction and quality assessment were allocated to one of the three pairs of researchers from the team for independent review. The research team as whole met and discussed after a pilot data extraction exercise using the draft form. The data extraction form was then finalised and the team met on a regular basis to ensure that data extraction and quality assessment across the pairs were consistent. Any disagreements were resolved through discussion.

### Synthesis

A narrative synthesis of the results was conducted. Due to the methodological nature of the systematic review, a meta-analysis was not considered appropriate.

### Outcome measures

The following outcomes were evaluated in the context of evaluation of minor ailment management: a) types of clinical outcomes used and b) methods of clinical outcome measurement.

### Guidelines considered for the review

Reporting of this review was based on the PRISMA statement and checklist [16] (S3 Appendix).

## Results

**Fig 1:**
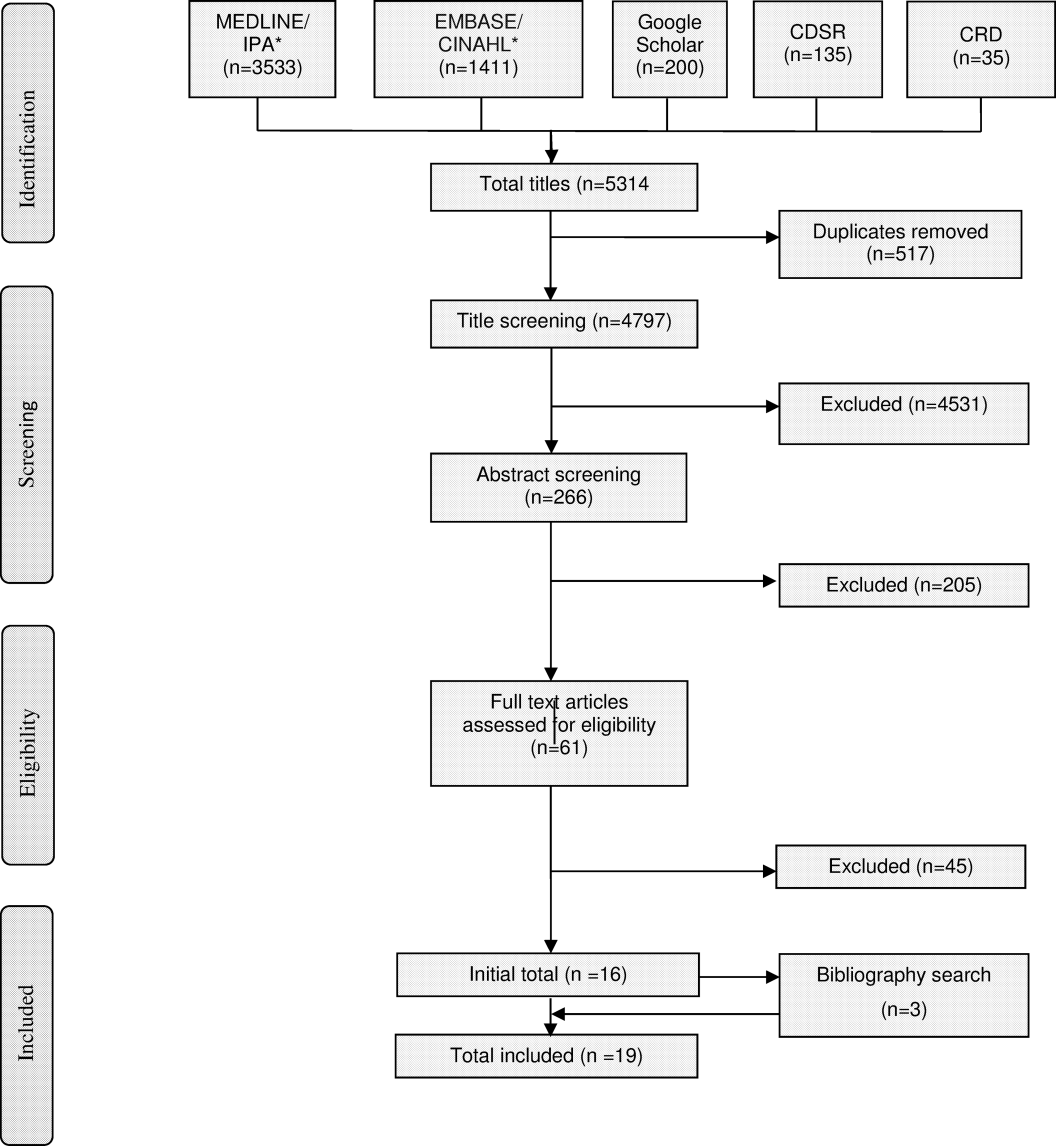
PRISMA flowchart of study selection process. *databases searched concurrently.

A total of 4798 unique titles were screened, of which 19 [1, 17–34] fulfilled eligibility criteria for inclusion in the review (Fig 1). All eligible papers were published in English language.

### Quality assessment

Among the 18 non-RCT studies assessed for quality using the CASP quality assessment tool, 17 studies presented clear study aims (Tables 1 and 2). The majority of studies did not provide adequate information on the recruitment strategy (n = 12). These included missing information on how the participants were identified and followed up. The majority of the included studies did not indicate whether the follow-up duration was validated in the context of clinical area being evaluated (n = 13). Confounding factors were only rarely considered when measuring the follow-up outcomes (n = 2).

**Table 1:** Quality assessment of included studies (non-RCTs).

Studies included	Did the study address a clearly focused issue?	Was the cohort recruited in an acceptable way?	Was the exposure accurately measured to minimise bias?	Was the outcome accurately measured to minimise bias?	Have the authors identified all important confounding factors?	Have the authors taken account of the confounding factors in the design and/or analysis?	Was the follow-up of subjects complete enough?	Was the follow-up of subjects long enough?	Are the results precise?
Bello (2013) (17)	Yes	Can't tell	Can't tell	Can't tell	Can't tell	No	Yes	Yes	Can't tell
Bosse (2012) (19)	Yes	Yes	Can't tell	Can't tell	Yes	No	Yes	Can't tell	Can't tell
Coelho (2014) (20)	No	No	Can’t tell	No	No	No	Yes	Can’t tell	Can't tell
Danno (2014) (21)	Yes	Can't tell	Can't tell	No	Yes	Can't tell	Can’t tell	Can't tell	Can't tell
Hacker (2012) (22)	Yes	Yes	Can't tell	Yes	Can't tell	Yes	Can’t tell	Can’t tell	Can’t tell
Klimek (2016) (23)	Yes	Can't tell	No	No	No	No	Can't tell	Can't tell	Can't tell
Krishnan (2000) (24)	Yes	Can't tell	Can't tell	Can't tell	No	Can't tell	Yes	Can't tell	Can't tell
Lambert (2013) (25)	Yes	Yes	Yes	Yes	Yes	No	Yes	Can't tell	Can't tell
Mansell (2015) (26)	Yes	Can't tell	Can't tell	No	No	No	Can't tell	Yes	Can't tell
Mehuys (2009) (27)	Yes	Yes	Yes	Yes	Yes	No	Yes	Can't tell	Can't tell
PANS (2013) (28)	Can't tell	Can't tell	Can't tell	No	No	No	Can't tell	Can't tell	Can't tell
Plunkett (2001) (29)	Yes	Can't tell	No	Can't tell	No	No	No	Can't tell	Can't tell
Schulz (2006) (30)	Yes	Can't tell	Can't tell	Can't tell	No	No	Can't tell	Can't tell	Can't tell
Sinclair (2001) (31)	Yes	Can't tell	Can't tell	Can't tell	No	No	Can't tell	Yes	Can't tell
Taylor (2017) (34)	Yes	Can't tell	Can't tell	Can't tell	No	Can't tell	Can't tell	Can't tell	Can't tell
Watson (2015) (1)	Yes	Yes	Yes	Yes	Yes	Yes	Yes	Yes	Can’t tell
Westerlund (2003) (32)	Yes	Yes	Can't tell	Can't tell	Yes	No	Yes	Can't tell	Can't tell
Whittington (2001) (33)	Yes	Can't tell	Can't tell	Can't tell	Yes	Can't tell	Can't tell	Can't tell	Can't tell

**Table 2:** Quality assessment of RCT (18) included in the review.

Quality assessment criteria	Result of quality assessment Birring (2017) (18)
Did the trial address a clearly focused issue?	Yes
Was the assignment of patients to treatments randomised?	Yes
Were all of the patients who entered the trial properly accounted for at is conclusion?	Yes
Were patients, health workers and study personnel 'blind' to treatment?	No
Were the groups similar at the start of the trial?	Can’t tell
Aside from the experimental intervention, were the groups treated equally?	Yes
Can the results be applied in your context (or to the local population)?	Can't tell
Were all clinically important outcomes considered?	Yes
Are the benefits worth the harms and costs?	Can't tell

The only RCT study (18) included in the study was single blinded at participant level. Information on the randomisation process was presented clearly. Comparative data on the baseline characteristics across the control and intervention groups however was not made available (Table 2). Complete outcome data were reported alongside intention to treat analysis.

### Study design and setting

One study utilised an RCT design [18] and the remainder (n = 18) used observational designs (Table 3). All included studies involved community pharmacy setting for which the clinical outcomes were measured [1, 17–34]. Other settings, such as general practice [1, 18, 32] and EDs [1], were also included as comparators in measuring effectiveness against pharmacy-based management of minor ailments (Table 3). One study was carried out with pharmacy service users as referred by an out-of-hours telephone helpline for non-life threatening conditions (National Health Service (NHS) Direct) [25].

**Table 3:** Characteristics of included studies.

Study	Year of study	Country	Study aim	Study design	Study setting (n)
Bello (2013) (17)	2011–13	Nigeria	To assess the impact of rural community pharmacist interventions on self-medications and disease prevalence	Observational	Community Pharmacy (1)
Birring (2017) (18)	2014/15	UK	To investigate the efficacy of CS1002, an OTC cough medicine for cough associated with upper respiratory tract infection, in a randomised controlled trial	RCT	General Practice (4) Community Pharmacies (12)
Bosse (2012) (19)	2009/10	USA	To evaluate the impact of community pharmacists on appropriate and successful use of available self-care treatment options	Observational	Community Pharmacies (2)
Coelho (2014) (20)	2012	Portugal	To determine the prevalence of self-medication and to evaluate the clinical impact of pharmaceutical counselling	Observational	Community Pharmacies (1)
Danno (2014) (21)	2010/11	France	To describe the socio-demographic and clinical characteristics of patients who seek direct therapeutic advice from a pharmacist for influenza-like illness or ear, nose and throat disorders, the types of medicines dispensed and patient satisfaction with the advice received	Observational	Community Pharmacies (133)
Hacker (2012) (22)	2012/13	Germany	To investigate: characteristics of gastrointestinal symptoms and patients’ global health status, drug usage and symptom relief, and patient satisfaction with the medication	Observational	Community Pharmacies (137)
Klimek (2016) (23)	2014/15	Germany	To explore factors affecting efficacy of treatment of common cold symptoms with an over-the- counter ibuprofen/ pseudoephedrine combination product	Observational	Community Pharmacies (230)
Krishnan (2000) (24)	1997/98	Germany	To assess the outcomes of self-medication and pharmacist's counselling in patients and demonstrate the value of pharmacist involvement in self-medication process	Observational	Community Pharmacies (36)
Lambert (2013) (25)	2009	UK	To determine financial and quality of life impact of patients calling the NHS Direct telephone helpline from the perspective of NHS service providers.	Observational	Community Pharmacies (unclear), Walk-in centre (unclear), 999 calls (unclear), general practice (unclear), ED (unclear), dentist (unclear), do nothing/treat at home (unclear)
Mansell (2015) (26)	2012/13	Canada	To determine whether patients prescribed minor ailment treatment by a pharmacist symptomatically improve within a set time frame.	Observational	Community Pharmacies (90)
Mehuys (2009) (27)	2007	Belgium	To investigate self-reported efficacy and frequency of use of OTC medication for minor complaints.	Observational	Community Pharmacies (63)
PANS (2013) (28)	2012/13	Canada	Unclear	Observational	Community Pharmacies (27)
Plunkett (2001) (29)	1997	Australia	To evaluate whether consumers were satisfied with advice about skin conditions received from community pharmacists and to estimate costs and potential savings to the consumer and government.	Observational	Community Pharmacies (126)
Schulz (2006) (30)	2003	Germany	To evaluate the 'real life' behaviour of consumers with non-prescription access to ambroxol hydrochloride cough syrup with special focus on tolerability and the pattern of product usage.	Observational	Community Pharmacies (266)
Sinclair (2001) (31)	1999	UK	To describe the recruitment rate, follow-up rates and level of symptoms improvement with pharmacy users of ibuprofen compared with non-users.	Observational	Community Pharmacies (61)
Taylor (2017) (34)	2015/16	Canada	To evaluate clinical outcomes in those receiving pharmacy-based care for 17 minor ailments.	Observational	Community Pharmacies (40)
Watson (2015) (1)	2009	UK	To compare health-related and cost-related outcomes of consultations for symptoms suggestive of minor ailments in EDs, GPs and community pharmacies.	Observational	Community Pharmacies (10) General Practices (6) EDs (2)
Westerlund (2003) (32)	2002	Sweden	To investigate the outcomes of a counselling model designed to help pharmacists care for customers seeking non-prescription treatment for dyspepsia in community pharmacies.	Observational	Community Pharmacies (6)
Whittington (2001) (33)	1999	UK	To describe community pharmacy management of minor conditions after referral from one general practice.	Observational	General practice (1) Community Pharmacies (8)

### Types of minor ailments

Studies either focused on one therapeutic area such as cough [18, 30], cold [23], skin conditions [29], gastrointestinal problems [22, 24, 27, 32] or multiple minor ailment areas [1, 17, 20, 21, 26, 28, 33, 34] (Table 4).

**Table 4:** Minor ailments evaluated, participants and sample sizes of the included studies.

Study	Minor ailment(s)	Describe the intervention delivered	Describe who delivered the intervention at each setting?	Participants	Sample size
Bello (2013) (17)	General body pain, headache, fever low back pain, osteoarthritis	Drug information and counselling on appropriate health management strategies for dyspepsia at baseline and bi-monthly for eighteen months	Community pharmacist	Unclear	Baseline: 730 Follow-up: Unclear
Birring (2017) (18)	Cough	Participants were randomised to CS1002 (Unicough) or simple linctus (SL), treatment duration was 7 days or until resolution of cough	GP, pharmacist in a general practice or community pharmacy. Participants self-administered their assigned medication	Aged ≥18 years who self-referred themselves to a GP or pharmacist with an acute cough of <7 days duration Severity of at least 60 mm on a 0–100 mm Visual Analogue Scale	Baseline: 163 Follow-up: 159
Bosse (2012) (19)	Unclear	Counselling and advice on self-care/ OTC medicines	Community pharmacists or pharmacy students under supervision of pharmacist	Aged ≥18 years who came into the pharmacy seeking self-care advice	Baseline: 72 Follow-up: 63
Coelho (2014) (20)	Minor ailments related to digestive, respiratory, dermal, nervous system, bone/muscle fever, asthenia, avitaminosis, ocular gynaecologist, vascular sytems.	Participants’ minor health problem were assessed and eligible participants targeted for pharmaceutical counselling, pharmacological and non-pharmacological treatments	Community pharmacist	Customers with at least one minor symptom or directly asking for a non-prescription medicine for their personal use	Baseline: 298 Follow-up: 268
Danno (2014) (21)	Influenza-like illnesses and ear, nose and throat disorders	Pharmacist conducted symptoms and medication history taking followed by recommendation of medicines and/or advice	Pharmacist	Early symptoms of an influenza-like illnesses or ear, nose or throat disorder that had appeared <36 h prior to the pharmacy visit; state of health not requiring a medical consultation; receiving at least one medication from the pharmacist (without a medical prescription); aged ≥12 years	Baseline: 573 Follow-up: unclear
Hacker (2012) (22)	Upper gastrointestinal symptoms	Sales of a specific antacid drug containing hydrotalcite as active ingredient.	Pharmacy staff	Unclear	Baseline:548 Follow-up: Unclear
Klimek (2016) (23)	Common cold	Sales of ibuprofen and pseudoephedrine combination product to pharmacy customers	Unclear	Aged ≥18 years purchasing ibuprofen and pseudoephedrine combination product	Baseline:1770 Follow-up: Unclear
Krishnan (2000) (24)	Dyspepsia	Patients in the intervention group received extensive questioning on factors associated with dyspepsia, medication counselling and instructions on dietary regulation and posture. Patients in the control group continued to receive the standard care provided by their pharmacist	Pharmacist	Patients who requested help for dyspepsia or asked by name for medication for dyspepsia	Baseline: 205 Follow-up: Unclear
Lambert (2013) (25)	Unclear	Participants managed by pharmacist after a triage through NHS Direct telephone helpline	Pharmacist	NHS Direct telephone callers who had consented to be contacted about their experience of the service.	Baseline: 3000 Follow-up: 1001
Mansell (2015) (26)	Acne, cold sores, diaper rash, canker sores, seasonal allergies, oral thrush insect bites	Pharmacist prescription of an eligible agent for minor ailment	Community Pharmacist	‘Adults’ prescribed an agent by a pharmacist for an applicable condition.	Baseline:125 Follow-up: 125
Mehuys (2009) (27)	Upper gastrointestinal symptoms	The pharmacist made a refer-or-treat decision, following a counselling protocol based on the Rome III criteria. Pharmacist then advised self-treatment options for patients using pharmacological or non-pharmacological advice	Pharmacy students under supervision of pharmacist	Pharmacy customers seeking self-medication for upper GI symptoms, aged 18–80 years, speaking Dutch, and agreeing to pay a follow- up visit to the pharmacy after 4 weeks	Baseline: 592 Follow-up: 566
PANS (2013) (28)	Various with Herpes simplex and allergic rhinitis as the most commonly managed	Pharmacist conducting a detailed assessment of the patient and making a prescribing decision; establishing a plan for follow-up with the patient and conducting follow-up as required; and following up as needed with the patient’s primary care provider	Not specified	Unclear	Baseline:1002 Follow-up: 871
Plunkett (2001) (29)	Inflammatory or infective skin conditions	Participants underwent dermatological consultations: diagnosis followed by product sales	Community pharmacist	Unclear	Baseline: 181 Follow-up: Unclear
Schulz (2006) (30)	Cough	Sales of a specific brand of cough medicines from pharmacies from those who requested the product or presented with symptoms	Unclear	Adolescent consumers who requested and bought a specific brand of ambroxol hydrochloride cough syrup	Baseline: 2707 Follow-up: Unclear
Sinclair (2001) (31)	Ibuprofen use for a range of minor ailments (not listed)	Ibuprofen tablet or capsule sales from community pharmacy	Unclear	Aged >17 years, able to give informed consent, and who purchased themselves (or had purchased on their behalf) a tablet or capsule form of ibuprofen	Baseline:555 Follow-up: 522
Taylor (2017) (34)	Acne, allergic rhinitis, athlete’s foot, canker sore, cold sore, diaper rash, dysmenorrhea, eczema, folliculitis headache, heartburn, haemorrhoids, impetigo, jock itch, sprain, ringworm, oral thrush	Pharmacy-based care (prescription of eligible agents) of minor ailments	Pharmacist	Adults prescribed an agent by a pharmacist for an applicable condition. If the medicine was for a child, a parent could participate	Baseline: 48 Follow-up: Unclear
Watson (2015) (1)	Musculoskeletal pain; eye discomfort; gastrointestinal disturbance; upper respiratory tract-related	Consultation of patients with the pharmacist or health care professional in pharmacies, general practices and EDs including diagnosis, counselling, advice and medicines provision	Pharmacist or healthcare professionals in general practice and EDs	Aged ≥18 years; requested treatment or medicines for one or more of the four included minor ailments or presented symptoms associated with these ailments; presented during specified times during the day and had face to face consultation with the staff	Baseline:377 Follow-up: 277
Westerlund (2003) (32)	Dyspepsia	Counselling of participants based on a counselling model, followed by provision of self-medication advice and referrals to physicians	Community pharmacist	Aged ≥18 years who asked for a advice or over the counter treatment for dyspepsia	Baseline: 319 Follow-up: 130
Whittington (2001) (33)	Constipation, cough diarrhoea, dyspepsia earache, hay fever head lice, headache high temperature, nasal symptoms, sore throat, vaginal thrush, URTI	Participant consultation with community pharmacist involving prescribing, from specific formulary, for minor ailment where necessary	Community pharmacist	Unclear	Baseline: 576 Follow-up: Unclear

### Participants, sample size and recruitment

Sample size calculations or sampling strategies were rarely justified [1, 18, 20] (Table 4). Inclusions were typically stipulated with regard to patient age (e.g. over 17/18 years) and presenting with the minor ailment subject to study or receiving treatment for a specific minor ailment.

### Intervention

The majority of studies stated that pharmacists or members of pharmacy staff delivered the intervention [17, 18, 20, 21, 24, 26, 29, 32–34]; although there was no clear distinction of their remit. One study used NHS Direct telephone staff to refer potential participants to out-of-hours pharmacy settings including referral to other settings such as the general practice [25]. Interventions were also delivered by general practitioners (GP) or nurses in a general practice [1, 18], and doctors or nurses in ED [1] with the general practice or ED used as a comparator setting against community pharmacy in the two studies [1, 25].

Nine studies reported that specific training had been provided to staff as either a component of the intervention or the research [19, 20, 24, 26–29, 31, 32]. For the majority of studies (n = 13), it was not specified who paid for the service at the point of care [1, 17–21, 24–28, 32, 34]. The remaining studies reported that minor ailment treatment was either paid for by patients [22, 23, 29–31, 33] or free at the point of care [33].

### Types of clinical outcomes used

#### Symptoms status

Clinical outcomes were evaluated in relation to symptom severity, pattern (i.e. frequency of specific symptoms) and resolution of symptoms (Table 5). Four studies evaluated disease-specific symptoms (Table 5). These included evaluation of associated symptoms of cough [18], influenza-like illness or ear, nose and throat disorders [21], cold [23] and gastrointestinal issues [27]. In contrast to the aforementioned, one study utilised a generic tool to gather data on the symptom status across four minor ailments at baseline and follow-up [1].

**Table 5:** Types of clinical outcomes and methods used for the measurement of clinical outcomes.

Study	Baseline clinical outcomes	Baseline data collection method (personnel responsible)	Follow-up clinical outcomes	Follow-up data collection (personnel responsible)	At what point follow-up data was captured?
Bello (2013) (17)	Prevalent illnesses (dyspepsia) and clinical data such as body temperature, height, weight, blood pressure	Questionnaire (self-administered or assisted)	Unclear	Unclear	Unclear
Birring (2017) (18)	Cough severity, frequency, sleep quality disruption in the previous 24 hours	Daily diary (self-administered)	Change of cough severity from baseline to day 4, 6 and 8 in cough severity on a VAS Time to resolution of cough symptoms VAS.	Daily diary (participant self-administered)	Daily entries from days 2–8.
Bosse (2012) (19)	Not available	Not available	Resolution of symptoms	Telephone survey (researcher)	1 week after consultations
Coelho (2014) (20)	Reason for the consultation	Face-to-face interview (pharmacist)	Symptom improvement	Face-to-face or telephone interview (pharmacist)	After 1 week.
Danno (2014) (21)	Intensity of 13 listed symptoms of influenza like illness and impact of these symptoms on sleep and daily activities using a global score; impact on the ability to carry out daily activities and sleep was assessed using a 7-point Likert scale	Questionnaire (self-administered)	Intensity of symptoms; any concomitant respiratory pathologies; adherence to the recommended treatments; use of any other treatments; impact of the illness on sleep and daily activities; and satisfaction with pharmacy service	Telephone interview (researcher)	3–5 days after inclusion
Hacker (2012) (22)	Symptoms (heartburn, acid regurgitation, epigastric pressure/pain, feeling of fullness, and others) and corresponding symptom severity on a four point scale from ‘non-existent’ to severe, global health status	Questionnaire (self-administered)	Symptoms along with corresponding symptom severity on a four point scale, effectiveness and side effects using treatment satisfaction questionnaire for medication	Questionnaire (participant self-administered)	Participants recorded their symptoms at 6 predefined time points (5, 10, 15, 30, 60, and 90 min) after medicines intake.
Klimek (2016) (23)	Four most bothersome cold symptoms rated on a 10 point scale; time from start of cold to first dose and to number of tablets at each dosing on the first, second, third or fourth day of treatment, if applicable	Questionnaire (self-administered)	Time to onset of symptom resolution; the extent of the 11 symptoms after the first dose of medication on a 10 point scale; duration of symptom relief after the first dose Participant responses to four disease-relevant statements; the tolerability of ibuprofen and pseudoephedrine combination product	Unclear	Unclear
Krishnan (2000) (24)	Gastrointestinal Quality-of-Life Index	Questionnaire (unknown)	Gastrointestinal Quality-of-Life Index	Questionnaire (unknown)	One week after initial visit to pharmacy
Lambert (2013) (25)	VAS quality of life (EQ-5D), health status	Telephone survey (researcher)	VAS quality of life question as included in the EuroQol-5D (EQ-5D), health status	Telephone survey (researcher)	Four to six weeks after initial call.
Mansell (2015) (26)	Not available	Not available	Symptom improvement and side effects	Questionnaire (participant self-administered)	Either 7 days (e.g. cold sores or insect bites) or 30 days (e.g. seasonal allergies) after having the prescription filled
Mehuys (2009) (27)	BMI, nature of GI symptoms e.g. heartburn; alarm symptoms e.g. weight loss, vomiting frequency and duration of the complaints, medical consultation, and medication use over the previous 12 months.	Questionnaire (self-administered)	Symptom resolution, whether medicines being taken currently and adherence to advice.	Questionnaire (participant self-administered) and participant medication diary	Four weeks after first pharmacy visit.
PANS (2013) (28)	Not available	Not available	Symptom resolution	Survey (unclear)	Unclear
Plunkett (2001) (29)	Diagnosis, as made by the pharmacist; OTC products recommended.	Questionnaire (pharmacist administered)	Symptoms resolution and perceived following of pharmacists’ advice.	Telephone survey (researcher)	Two to six weeks after consultation
Schulz (2006) (30)	Pattern of symptoms including frequency of cough events in the last 12 months, mean duration of an event and current pattern	Questionnaire (self-administered)	Symptoms resolution, and tolerability and re-consultation with a physician.	Questionnaire (participant self-administered)	Seven days
Sinclair (2001) (31)	Not available	Not available	Self-reported information on ibuprofen usage, reason why the drug was purchased, concurrent medication, symptoms experienced and health service utilisation.	Questionnaire (participant self-administered)	After one week and 2, 6 and 12 months
Taylor (2017) (34)	Not available	Not available	Symptom improvement, side-effects and efficacy of agent.	Questionnaire (participant self-administered)	Either 7 day point (e.g. cold sores/oral thrush) or 30 day point (e.g. seasonal allergies)
Watson (2015) (1)	Quality of life (EQ-5D/EQ-VAS), perceived seriousness and duration of symptoms.	Questionnaire (self-administered)	Symptom resolution, quality of life, re-consultation for the index ailment and health service utilisation since their index consultation and quality of life (EQ-5D).	Questionnaire (participant self-administered)	Post-consultation and at 2 weeks.
Westerlund (2003) (32)	Common symptoms	Interview (method unclear)	Dyspepsia symptoms resolution, drug related problems, re-consultation with a physician	Interview (method unclear)	Pharmacy customers: 1–2 weeks after pharmacy visit; referred customers: 3/4 weeks after pharmacy visit
Whittington (2001) (33)	Minor condition(s) dealt with, whether a prescription was dispensed with item and quantity, whether an OTC product was purchased or whether the patient was referred back to the practice	Study form (pharmacist administered)	Re-consultation with GP following pharmacist consultation.	Unclear	Unclear

*tools mentioned where available

Various methods were used to gather clinical data on symptom severity. These included a Visual Analogue Scale (VAS) [27] and Likert scales ranging from four [21, 22] to 11 points [23]. One study reported using body mass index (BMI) measures, as a means of detecting symptoms which were associated with more serious conditions, in addition to measuring minor ailment specific symptoms [27].

#### Re-consultation

Re-consultation was assessed in four studies and was intended as a surrogate follow-up measure of clinical outcome assessment [1, 30, 32,33]. Patients in these studies were asked whether they had subsequently consulted with a GP/physician for the minor ailment after their index consultation. In addition, one study also evaluated re-consultation within the same setting for the minor ailment presented during the index consultation [1].

#### Adverse clinical outcomes

Only seven studies included in this review evaluated adverse clinical outcomes (Table 5). A lack of standardised terminology and data collection tools was observed in relation to referrals and reporting adverse clinical outcomes. Terminologies used to refer to adverse clinical outcomes included: side effects, tolerability, drug-drug interactions, drug related problems (DRPs), overuse and misuse. For example, tolerability was a follow-up outcome in two studies evaluating the clinical outcomes of pharmacy management of cough [30] and cold [23]. Tolerability for the study purpose was not defined in either study. In general, there was a lack of information provided on the validation of tools utilised in the pharmacy setting.

#### Quality of life and health status

Four studies evaluated quality of life outcomes, two of which used disease-specific tools [18, 24]. Birring [18] used the Leicester Cough Questionnaire for acute cough at baseline and follow-up. Krishnan [24] used Gastro-intestinal Quality of Life Index in the assessment of outcomes of patients recommended treatment for dyspepsia at baseline and follow-up. Neither study provided information on the validation of the tools or whether they were fit for use in the pharmacy setting. Two studies used more generic tools, for example, the Generic EuroQoL was used to evaluate quality of life in two studies [1, 25] at both baseline and follow-up. These studies did not justify whether the tools used were sensitive or had been validated for use in the context of minor ailments. Health status was evaluated using global measures in four studies [1, 22, 25, 26].

### Methods of clinical outcomes assessment

#### Data collection tools and their administration

Scant methodological details were reported around how baseline and/or follow-up clinical data were collected from study participants (Table 5). Five studies described that baseline clinical data were recorded by the pharmacist or member of pharmacy staff in the pharmacy setting, as a component of the clinical consultation using structured questionnaires [17, 27–29, 33]. Four studies collected baseline data via self-administered or researcher-administered questionnaires in pharmacy or comparator settings [1, 17, 23, 33].

A limited number of studies attempted to provide information about the validation of the methods used to collect either baseline or follow-up clinical data (Table 5). Where information was available on the development of the data collection tools, these were mostly limited to testing of face and content validity. The utility of generic validated quality of life instruments within the context of the clinical areas evaluated was not clear [1, 25].

#### Baseline and follow-up data

Five studies did not collect any baseline data [19, 24, 28, 31, 34] (Table 5). Two studies were explicit in reporting the timing of administration of data collection tools: either prior to [1] or after the index consultations [26]. Various timelines were used to evaluate follow-up clinical data. The majority of studies used a single time point for the evaluation of follow-up data with the exception of three studies where multiple time points were used [18, 26, 31]. Follow-up timelines were specific to each minor ailment in one study [26]. To illustrate, timelines were either 7 days for cold sores or insect bites, and 30 days from the index consultation for seasonal allergies based on the length of the symptoms [26]. Symptom or medication diaries were used by patients in two studies [18, 27].

#### Missing follow-up data

Statistical or methodological techniques used to extrapolate follow-up missing data were applied by one study [22]. This study used the ‘last observation carried forward’ technique as imputation method for missing data over time.

#### Patient satisfaction measures

This systematic review only sought to review the methods used to measure patient satisfaction if they were assessed alongside clinical outcomes. Ten studies did not measure patient satisfaction [17–20, 24, 25, 27, 30, 31, 33]. Studies which utilised patient satisfaction measures varied in their approach, either using categorical questions [23, 26, 32], Likert scales [26, 29, 34], the Treatment Satisfaction Questionnaire for Medication [22] or the Medical Interview Satisfaction Scale [1]. One study did not clarify how patient satisfaction was measured [28]. Two studies reported using validated tools for measuring patient satisfaction [1, 22].

#### Results related to clinical outcomes

Improvement of clinical outcomes was reported across a number of studies [1, 17–28, 30, 32]. Two studies demonstrated that the clinical effectiveness of pharmacy-based minor ailment management was equivalent to, or improved, when compared with management by other health care professionals in settings such as general practice or EDs [1, 25].

## Discussion

This study aimed to systematically review the methods and types of clinical outcomes in the evaluation of pharmacy-based management of minor ailments. The results have demonstrated a lack of high quality, adequately powered studies used in the evaluation of pharmacy-based minor ailments management. Amongst the studies included, only one study used an RCT design and there was a lack of adequately powered longitudinal follow-up studies. Within the included studies, explicit adherence to best practice guidelines [35–38] relating to study methodology was notably lacking. The Medical Research Council Framework signifies the importance of developing and validating the methodological tools used in the developing and evaluation of complex interventions [39].

The included studies evaluated a range of clinical outcomes which included symptom status (inclusive of symptom severity, pattern, and resolution); re-consultation; adverse clinical outcomes; and quality of life. However, key methodological information was missing around the choice of clinical outcomes used, the development and validation of data collection tools used and the timelines of baseline and follow-up outcome data. Given that some of the minor ailments may be self-limiting in nature, lack of validation of the follow-up timeline may affect estimates of the impact of the interventions. A number of studies evaluated the clinical outcome data only at the follow-up stages.

A number of studies did not utilise disease-specific clinical outcome assessments. Given the diverse clinical areas being evaluated, there is scope for the development of core outcome sets that could be applied to the evaluation of a range of minor ailments. Such core outcome sets have been developed through consensus around reporting of clinical outcomes in clinical trials [40]. Consistent outcome sets would also facilitate the ability to conduct meta-analyses of clinical outcomes and direct comparisons between studies.

A minority of the included studies considered evaluation of adverse clinical outcomes data at follow-up and these were often poorly defined in study reports. Best practice guidelines suggest that study reports include objective information on the incidence and type of clinically relevant adverse events (including any adverse events requiring discontinuation of therapy) as opposed to the use of general statements such as “well tolerated”. Information on the severity, frequency, and timing of adverse events are of additional value [40]. Various types of symptom severity scales were used in the included studies, for both positive clinical outcomes and the adverse clinical outcomes. In particular, there was a lack of information provided on the validation of tools in the context of their use in minor ailments and pharmacy as a study setting. Study settings and disease conditions can have important influence in the validity and reliability of symptoms and quality of life assessment tools [41]. Future research should focus on developing standard practice to address the variance in their use.

### Strengths and limitations

This is the first methodological systematic review of studies evaluating the type of clinical outcomes and methods used for their assessment in the evaluation of pharmacy-based management of minor ailments. Standard guidelines [14, 15] were used to inform the review process. Being a methodological systematic review, this study did not seek to review in-depth the impact of pharmacy management of minor ailments on clinical outcomes. This systematic review applied wider inclusion criteria when considering the selection of studies. Thereby, the studies included in the review evaluated a broad range of minor ailments. Hence, in-depth consideration of methodological aspects of research relevant to individual clinical areas were not possible.

### Impact on practice

The lack of high quality research, in terms of methodological rigour, as identified by this systematic review, is a barrier to the promotion of pharmacy-based services aimed at minor ailments management. In the UK, despite the country leading the reclassification of prescription only medicines for over-the-counter supply (including pharmacy only status), the burden of minor ailments in high cost settings still remains a key issue. This review demonstrates a lack of high quality evidence in relation to the clinical outcomes of pharmacy-based management of minor ailments; a factor which may contribute to failings to shift care from high cost settings, such as EDs and general practices. Reclassification decisions are often based on the ‘do no harm’ principle and on the experiences of their use as a prescription medicine and assumption of reduced costs to the health services. The evidence base for pharmacy-based provision of services and medicines are often ignored in such decisions.

### Recommendations for research

The results of this study suggest that future research on pharmacy-based management of minor ailments should adhere to the following recommendations:

- Utilisation of high quality RCT or longitudinal observational studies informed by best practice guidelines.
- Use of validated clinical outcome measures to generate high quality evidence. These include development and use of disease-specific outcomes assessment tools and core-outcome sets for a range of minor ailments.
- Use of out-of-hours services, online healthcare services, self-care without professional support, and over-the-counter management in non-pharmacy setting, in addition to pharmacist-led services in general practices and ED comparators to community pharmacy-based models of minor ailments management. Comparative evaluations will be of value to practitioners, policy makers and researchers in terms of identifying service improvement and cost effectiveness.
- Research studies should distinguish between interventions delivered by pharmacists and those delivered by support staff under pharmacist supervision in a pharmacy.
- Inclusion of adverse clinical outcomes since they were rarely considered by the included studies and are particularly important where clinical areas are novel such as in the context of the evaluation of newly reclassified medicines aimed for pharmacy supply.

## Conclusions

Currently, there are methodological limitations in the studies that have sought to evaluate clinical outcomes of pharmacy-based management of minor ailments with regard to both type and method of assessing clinical outcomes. Future evaluations of pharmacy-based management of minor ailments should consider the use of high quality study designs, informed by best practice methodological guidelines, and validated methods of measuring clinical outcomes. There is scope for development of a core outcomes set specific to minor ailments management and development of a validated methodology for measuring such outcomes in a research study.

## Supporting information

S1 Appendix Published protocol. (DOCX)

S2 Appendix Search strategy. (DOCX)

S3 Appendix PRISMA checklist. (DOCX)
