# Supplementary material for: Methodological considerations in clinical outcomes assessment of pharmacy-based minor ailments management: A systematic review
Source: PLoS One. 2018 Oct 4;13(10):e0205087. doi: 10.1371/journal.pone.0205087 (PMC6171901; doi:10.1371/journal.pone.0205087)
Supplement: S1 Appendix — (DOCX) [file pone.0205087.s001.docx]

Appendix 1: Published protocol

[[
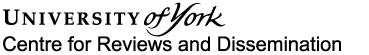
](http://www.york.ac.uk/inst/crd/)](http://www.york.ac.uk/inst/crd/)[[
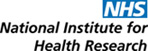
](http://www.nihr.ac.uk/)](http://www.nihr.ac.uk/)

**PROSPERO International prospective register of systematic reviews**

| \| \|  \| \| --- \| \| **Types of clinical outcomes and methods for their assessment in the evaluation of pharmacist-led management of minor ailments: a systematic review** \| \| *Vibhu Paudyal, Scott Cunningham, Katie MacLure, Katie Gibson Smith, Cristin Ryan, Maria Cordina* \| \|  \| \| **Citation**  Vibhu Paudyal, Scott Cunningham, Katie MacLure, Katie Gibson Smith, Cristin Ryan, Maria Cordina. Types of clinical outcomes and methods for their assessment in the evaluation of pharmacist-led management of minor ailments: a systematic review. PROSPERO 2016:CRD42016050847 Available from <http://www.crd.york.ac.uk/PROSPERO/display_record.asp?ID=CRD42016050847> \| \| **Review question(s)**  1. To systematically review the types of clinical outcomes used in the evaluation of pharmacist-led minor ailments management.  2. To systematically review the methods of clinical outcomes assessment used in the evaluation of pharmacist-led minor ailments management.  3. To inform development and application of best practices regarding the types and methods of clinical outcomes assessment in the evaluations of pharmacist-led minor ailments management.  **Searches**  1.MEDLINE  2.EMBASE  3.CINAHL  4.International Pharmaceutical Abstracts (IPA)  5.Cochrane Database of Systematic Reviews (CDSR)  6.Google Scholar  7.Centre for Review and Dissemination (CRD) database  The bibliography of included studies will be reviewed to further identify additional references. Relevant international experts will be contacted by email to seek additional studies.  Boolean operators such as truncations (*), wild cards ($), adjacent search options (e.g. adj2) will be used where relevant. Refworks will be used to manage the search results.  **Types of study to be included**  There will be no restriction on the study designs included, for example any randomized studies, cohort studies and before and after evaluations.  **Condition or domain being studied**  Minor ailments  **Participants/ population**  There will be no limitations to study populations. Both children and adults will be included.  **Intervention(s), exposure(s)**  Evaluations of pharmacy-led (including community and hospital) management including comparative evaluations of minor ailments management involving the following will be included:  • General Practice  • Out of hours (including Accident and Emergency)  • Walk-in centres  • Unscheduled care providers  • Self-care with no professional support including over the counter management in non-pharmacy settings.  With regards to community pharmacy management, evaluation of both publicly-funded minor ailments schemes, over-the-counter treatment, service provision through patient group directions will be included.  All minor ailment types will be included. An established typology (based on the list of minor ailments or the relevant pharmacy medicines) will be used to list the minor ailments included in the evaluation.  **Comparator(s)/ control**  Evaluations of pharmacy-led (including community and hospital) management including comparative evaluations of minor ailments management involving the following will be included:  • General Practice  • Out of hours (including Accident and Emergency)  • Walk-in centres  • Unscheduled care providers  • Self-care with no professional support including over the counter management in non-pharmacy settings.  With regards to community pharmacy management, evaluation of both publicly-funded minor ailments schemes, over-the-counter treatment, service provision through patient group directions will be included.  All minor ailment types will be included. An established typology (based on the list of minor ailments or the relevant pharmacy medicines) will be used to list the minor ailments included in the evaluation.  **Context**  Exclusion criteria  • Evaluation not involving clinical outcomes assessment.  • Literature based only on conceptual models, i.e. lacking empirical evidence.  **Outcome(s)**  **Primary outcomes**  • Types of clinical outcomes used in the evaluations of minor ailments management  • Methods of clinical outcomes assessment in the evaluations of minor ailments management e.g. follow up, patient assessment, methods to obtain self-reported data etc.  **Secondary outcomes**  Recommendations for development and implementation of best practices with regards to the using of the types and methods of clinical outcomes assessment for a range of minor ailments or specific minor ailment types  **Data extraction, (selection and coding)**  A data extraction tool will be developed based on the aims and objectives and will be piloted using two research papers. The following key data will be extracted:  • Details of the authors, country of publication/study, year of publication, study population, setting, patient identification and recruitment  • Details of any intervention including features of minor ailment schemes, comparator services  • Types of clinical outcomes used  • Measures of clinical outcomes assessment  • Any validation of clinical outcomes assessment  • Results, summary measures with p values and confidence intervals, where reported.  Independent data extraction of each included study will be undertaken.  **Risk of bias (quality) assessment**  An independent, duplicate quality assessment of each study will be undertaken. Validated tools will be used to perform critical appraisal such as those developed by the Critical Appraisal Skills Programme (CASP). Risk of bias assessment will be conducted for randomised controlled trials using the Cochrane Collaboration tool for assessing risk of bias. Studies will not be excluded based on quality criteria alone.  **Strategy for data synthesis**  The nature of aims and objectives will lend to a narrative synthesis of the results. Being a methodological systematic review, a meta-analysis is not warranted. Microsoft Excel will be used to manage the data extraction and quality assessment processes.  **Analysis of subgroups or subsets**  None planned  **Dissemination plans**  Peer reviewed journal publication, study report to the grant awarding body (European Society of Clinical Pharmacy), abstract presentation at relevant conferences.  **Contact details for further information**  Dr Paudyal  School of Pharmacy and Life Sciences, Robert Gordon University, Garthdee Road, Aberdeen, AB10 7GJ  v.paudyal1@rgu.ac.uk  **Organisational affiliation of the review**  Robert Gordon University  www.rgu.ac.uk  **Review team**  Dr Vibhu Paudyal, Robert Gordon University Dr Scott Cunningham, Robert Gordon University Dr Katie MacLure, Robert Gordon University Dr Katie Gibson Smith, Robert Gordon University Dr Cristin Ryan, Royal College of Surgeons in Ireland Professor Maria Cordina, University of Malta  **Anticipated or actual start date**  01 November 2016  **Anticipated completion date**  31 July 2017  **Funding sources/sponsors**  European Society of Clinical Pharmacy 2016 Grant  **Conflicts of interest**  None known  **Language**  English  **Country**  Scotland  **Subject index terms status**  Subject indexing assigned by CRD  **Subject index terms**  Community Pharmacy Services; Humans; Pharmacists; Referral and Consultation  **Stage of review**  Ongoing  **Date of registration in PROSPERO**  07 November 2016  **Date of publication of this revision**  08 December 2016   \| **Stage of review at time of this submission** \| **Started** \|  \| **Completed** \| \| --- \| --- \| --- \| --- \| \| Preliminary searches \| No \|  \| No \| \| Piloting of the study selection process \| No \|  \| No \| \| Formal screening of search results against eligibility criteria \| No \|  \| No \| \| Data extraction \| No \|  \| No \| \| Risk of bias (quality) assessment \| No \|  \| No \| \| Data analysis \| No \|  \| No \| \|  \| \| \| \| \| \|  \| \| PROSPERO This information has been provided by the named contact for this review. CRD has accepted this information in good faith and registered the review in PROSPERO. CRD bears no responsibility or liability for the content of this registration record, any associated files or external websites. \| \|  \| \| \| --- \| --- \| --- \| --- \| --- \| --- \| --- \| --- \| --- \| --- \| --- \| --- \| --- \| --- \| --- \| --- \| --- \| --- \| --- \| --- \| --- \| --- \| --- \| --- \| --- \| --- \| --- \| --- \| --- \| --- \| --- \| --- \| --- \| --- \| --- \| --- \| --- \| --- \| --- \| --- \| --- \| --- \| |  | \| **Options** \| \| --- \| \| Print \| \| PDF \| \|  \| \| **Share**  Share \| \|  \| \| Revision Notes \| \| Revision History \| \| \| Nov 7 2016 7:00AM \| \| --- \| \| |
| --- | --- | --- | --- | --- | --- | --- | --- | --- | --- | --- | --- | --- | --- | --- | --- | --- | --- | --- | --- | --- | --- | --- | --- | --- | --- | --- | --- | --- | --- | --- | --- | --- | --- | --- | --- | --- | --- | --- | --- | --- | --- | --- | --- | --- | --- | --- | --- | --- | --- | --- | --- | --- | --- | --- |
