# Supplementary material for: Methodological considerations in clinical outcomes assessment of pharmacy-based minor ailments management: A systematic review
Source: PLoS One. 2018 Oct 4;13(10):e0205087. doi: 10.1371/journal.pone.0205087 (PMC6171901; doi:10.1371/journal.pone.0205087)
Supplement: S2 Appendix — (DOCX) [file pone.0205087.s002.docx]

Appendix 2

MEDLINE/EMBASE literature search strategy and results

| **ID** | **Search term** | **Results** |
| --- | --- | --- |
|  | Pharmacy/or Pharmacy Service, Hopsital/ | 107603 |
|  | Pharmacy.mp. | 158845 |
|  | Limit 2 to abstracts | 86439 |
|  | Pharmacies.mp. | 26989 |
|  | Limit 4 to abstracts | 19321 |
|  | Pharmaceutical Services/ or Pharmacists/ or Community Pharmacy Services/ | 138036 |
|  | Pharmacist$.mp. | 105496 |
|  | Limit 7 to abstracts | 63990 |
|  | Retail clinic$.mp. | 251 |
|  | Limit 9 to abstracts | 153 |
|  | **Combine 1 to 10 with OR** | 206140 |
|  | Self Medication/ | 14360 |
|  | Minor ailment$.mp. | 602 |
|  | Minor illness$.mp. | 1040 |
|  | Minor injur$.mp. | 3718 |
|  | Non-urgent.mp. | 1762 |
|  | Common illness$.mp. | 1752 |
|  | Common ailment$.mp. | 687 |
|  | ((minor or common or self-limiting or non-urgent or nonemergency or non-emergency) adj3 (ailment or illness or sickness or symptom or injury or condition or problem)).mp. | 97333 |
|  | Limit 19 to abstracts | 95888 |
|  | Over-the-counter.mp. | 16275 |
|  | Limit 21 to abstracts | 14735 |
|  | Otc.mp. | 9930 |
|  | Limit 23 to abstracts | 8708 |
|  | Non-prescription.mp. | 13638 |
|  | Limit 25 to abstracts | 8641 |
|  | **Combine 12 to 26 with OR** | 137779 |
|  | **Combine 11 and 27** | 5631 |
|  | **Limit 28 to yr=”2000-Current”** | 4632 |
|  | **Remove duplicates from 29** | 3533 |
